# Shall Nurses Cease to Be Laywomen?—IV

**Published:** 1891-10-03

**Authors:** 


### Passing Topics.

### SHALL NURSES CEASE TO BE LAYWOMEN?—IV.

(*Continued from page* 300, *Vol. X.*)

Next to the patients we can form a shrewd guess that physicians and surgeons would be the first to suffer by the advance of the nurse from the position of handmaid to colleague. If we are to credit the gathering testimony of a class of medical gentlemen we all know and respect—those engaged in family practice—very considerable friction already exists owing to the attitude adopted by many nurses fresh from their course of hospital training, towards those who, with the licence of medical students, they superciliously term "G. P.'s" The latter they honestly believe to be poor ignorant souls groping in mediæval darkness, unreached by a ray of true scientific light, and who ought to be grateful for the advent of a coadjutor brilliant with the reflected glory of a great medical school, and able to detect and set right any number of old-fashioned errors.

If we need proof that the danger is a real one, we have only to refer back to the record of the proceedings of the late Medical Congress at Bournemouth to find that whatever may be the opinions of those who have lent the weight of their names to the proposal for a "legally constituted profession of nursing," the great body of medical practitioners are keenly alive to its folly. "You may doubt," remarks a Lady Superintendent, who is addressing in the current number of the organ of registration, an imaginary class of probationers,—"You may doubt in your own mind the wisdom of an order," but—"yours is to obey." What must be the outcome of dalliance like this? If a probationer be encouraged to "doubt the wisdom" of the doctor's order, may not a "legally constituted" nurse—the member of a profession—the coadjutor of hospital physicians, feel justified in doing something more? We know what the answer of some nurses already is.

The argument that because doctors and lawyers are members of professions, therefore women who take to nursing are entitled to a professional status, overlooks many important considerations. The training of a lawyer or a doctor differs very materially from that of a nurse. Considerable erudition to start with, years of subsequent study, and a large expenditure of money are necessary in the former cases; in regard to the nurse, reading and writing are the only requirements actually indispensable, and the training not only costs her nothing, but board, lodging, uniform, and wages are given while it lasts. And this is not all. Nurses constitute the rank and file of their organisation; and there is no more injustice in asking nurses to sink the individual in their order, than in asking soldiers, policemen, sailors, clerks, and many other people to do the same. To all alike there may be a chance of reaching a higher grade, and the efficient nurse always possesses a prospect of promotion sufficient to satisfy a reasonable ambition.

Returning for a moment to the hospital standpoint, we may remark that a result of constituting nursing a profession would be to bring nurses into closer and more familiar relations with the house doctors and students, and it is one to be devoutly deprecated. The mixing of nurses and students is already a source of anxiety to Matrons and hospital authorities generally; but, at least, a nurse occupies a place in regard to the patient distinctly separate from that of the medical officers. There is no possibility of confounding the duties of the one with the duties of the other. A Sister or nurse is a power in her ward. If she please she may inspire respect and deference in breasts not commonly overburdened with these qualities, and she exercises a moderating influence upon the raw youths who fill the junior posts about the hospital. She owes this influence wholly to the fact that she is not one of themselves. As soon as the lay qualities of the nurse are impaired, her prerogatives will go, and her position will become approximated to that of a dresser. Agricola.

(*To be continued.*)

